# Greater endogenous pain facilitation is associated with lower spinal excitability and maximal knee extension strength deficits in athletes with patellar tendinopathy

**DOI:** 10.1007/s00421-026-06166-0

**Published:** 2026-02-25

**Authors:** Patrick Vallance, Dawson J. Kidgell, Peter Malliaras

**Affiliations:** 1 Monash Musculoskeletal Research Unit, Department of Physiotherapy, School of Primary and Allied Health Care, Faculty of Medicine, Nursing and Health Science, Monash University, Melbourne, Australia; 2 Monash Exercise Neuroplasticity Research Unit, Department of Physiotherapy, School of Primary and Allied Health Care, Faculty of Medicine, Nursing and Health Science, Monash University, Melbourne, Australia; 3 Department of Physiotherapy, School of Primary and Allied Health Care, Faculty of Medicine, Nursing and Health Science, Monash University, PO Box 527, Frankston, Melbourne, VIC 3199 Australia

**Keywords:** Patellar tendinopathy, Endogenous pain inhibition, Transcranial magnetic simulation, Electrical stimulation, Maximal voluntary isometric force, Strength, Pain

## Abstract

**Purpose:**

Recent discoveries indicate neurophysiological mechanisms might underlie pain symptoms and knee extension strength deficits in patellar tendinopathy. Specifically, reduced spinal excitability could underlie strength deficits, while a proportion of individuals exhibit altered endogenous pain modulation. Parallels can be drawn to other persistent knee conditions where links between pain and motor neurophysiological mechanisms have been established. Whether similar neural mechanism interplay occurs in patellar tendinopathy has not yet been investigated. We aimed to determine whether endogenous pain modulation was associated with motor neurophysiological outcomes in patellar tendinopathy.

**Methods:**

We included *n* = 19 athletes with patellar tendinopathy who participated in two cross-sectional studies; one assessing endogenous pain modulation, and the other motor neurophysiological function. We quantified pain inhibition as pressure pain threshold change immediately following 120-seconds hand immersion in painful cold-water, while pain facilitation was calculated as hand-pain change from five to 20 s. Following knee extension MVIF assessment, we used transcranial magnetic stimulation (TMS) to measure corticospinal excitability and inhibition via production of motor evoked potentials and their silent period. Additionally, we collected lumbar-evoked potentials (LEPs) to quantify spinal excitability. We could only collect TMS and LEP outcomes in *n* = 15 and *n* = 17 participants, respectively.

**Results:**

Greater pain facilitation predicted higher LEP (indicating lower spinal excitability) (*β* = 38.4 [95%CI 10.3–77.0]) and reduced strength (*β*=-7.9 [95%CI − 15.1 to − 2.6]). Pain inhibition was not associated with motor neurophysiological outcomes (*p* > 0.05).

**Conclusion:**

Our findings provide preliminary evidence of interplay between the neurophysiological mechanisms underlying pain facilitation and knee extension strength deficits in patellar tendinopathy.

**Supplementary Information:**

The online version contains supplementary material available at 10.1007/s00421-026-06166-0.

## Introduction

Patellar tendinopathy is a highly prevalent musculoskeletal condition, particularly among athletes who participate in jumping sports, e.g., 21% of basketball and 25% of volleyball athletes are affected, respectively (Nutarelli et al. 2023). It is defined by hallmark signs of pain that is localised to the tendon’s insertion at the inferior pole of the patella (Malliaras et al. 2015) and deficits in maximal knee extension strength (Obst et al. 2024). The condition is associated with substantial burden, with nearly one in five affected athletes retiring from their sport prematurely due to their symptoms, while it is also considerably persistent (Visnes et al. 2024). In particular, one prospective study reported no improvement in tendinopathy-specific disability - measured with the Victorian Institute of Sport Assessment–Patellar (VISA-P) questionnaire - compared to baseline at an 11-year follow-up (Visnes et al. 2024).

Given patellar tendinopathy does not appear to resolve without intervention (Visnes et al. 2024), the application of rehabilitation should be regarded as a necessary step to promote recovery. However, current rehabilitation approaches are only partially effective, as a proportion of individuals do not experience clinically meaningful improvement in pain, function or ability to play sport - as indicated by change in the VISA-P questionnaire (Breda et al. 2021). Specifically, 22–38% of athletes with patellar tendinopathy who underwent 24-weeks progressive tendon-loading exercise did not achieve the minimum clinically important improvement in VISA-P, when this outcome was calculated as change relative to their baseline score (Breda et al. 2021; Hernandez-Sanchez et al. 2014). This raises questions as to why some people with patellar tendinopathy do not experience clinically meaningful recovery with rehabilitation, while others do. One plausible explanation is that these rehabilitation protocols being utilised do not sufficiently target the specific neurophysiological mechanisms underlying pain symptoms and knee extension strength deficits in this condition.

Recent scientific advances have contributed to a more nuanced understanding of these mechanisms (Davi et al. 2020; Vallance et al. 2024, 2025a, b), yet rehabilitation protocols have remained largely static across the same period. For example, recent research revealed that knee extension strength deficits in individuals with patellar tendinopathy are likely to be mediated by spinal mechanisms. Specifically, this could be driven by increased activity of spinal interneurons which inhibit transmission between corticospinal axons and motoneurons (Davi et al. 2020; Vallance et al. 2024). Regarding mechanisms that could underlie pain symptoms in patellar tendinopathy, our research recently identified considerable individual variability in the efficacy of neural circuits that are responsible for endogenous pain inhibition and facilitation (Vallance et al. 2025a, b). Critically, this indicates that certain individuals with patellar tendinopathy might experience greater pain as a result of enhanced synaptic transmission of nociceptive afferents at the spinal dorsal horn, driven by supraspinal projections that alter synaptic efficiency (Yarnitsky et al. 2014). This variability suggests that the neurophysiological mechanisms driving symptoms in patellar tendinopathy might not necessarily be uniform. This is an important point, as it could provide some insight for poor rehabilitation response experienced by a number of individuals with this condition (Georgopoulos et al. 2019).

It is reasonable to speculate that the application of updated interventions to directly target the underlying neurophysiological mechanisms could produce improved rehabilitation outcomes. In particular, this might be most relevant to those individuals who are less responsive to contemporary protocols, who could potentially also represent the subset with greater neurophysiological mechanism alterations. Indeed, in certain persistent musculoskeletal conditions, greater alterations to neurophysiological circuits responsible for the modulation of pain – as measured via conditioned pain modulation and temporal summation protocols – have predicted worse treatment response (Georgopoulos et al. 2019). Moreover, it has been suggested that improved knowledge for such alterations might provide the opportunity for treatment that is more individualised (Bannister and Hughes 2023). In the context of patellar tendinopathy, the implementation of any such individualised approach is not yet possible, as this would first require a more comprehensive understanding of the specific neurophysiological mechanisms driving pain symptoms and functional impairments. If these can be better understood, it could pave the way for the design of rehabilitation that better serves individuals who are less responsive to current rehabilitation.

When recent evidence is considered in full, it provides insight for potential interplay between the neurophysiological mechanisms underlying pain symptoms and knee extension strength deficits (Vallance 2025). Similar overlap has been well-documented in other persistent musculoskeletal conditions affecting the knee region, including patellofemoral pain and knee osteoarthritis (Rice and McNair 2010). Specifically, this complex phenomenon - known as quadriceps artherogenic muscle inhibition (AMI) - represents a critical neuromuscular mechanism contributing to knee extension strength deficits (Rice and McNair 2010). Artherogenic muscle inhibition is thought to result from persistent and/or altered discharge of nociceptive and non-nociceptive afferents, which influence the excitability of motor pathways. In particular, synaptic transmission of nociceptive afferents at the spinal cord dorsal horn can excite inhibitory spinal interneurons, reducing the efficacy of efferent transmission between corticospinal inputs and motoneurons (Rice and McNair 2010). It is worth noting however that this mechanism is multifaceted. For example, it has been suggested that discharge of nociceptive afferents might also influence force output through effect on the function of neural elements further upstream (Rice et al. 2014).

While hypothetical theory can be generated for potential interplay between the neurophysiological mechanisms underlying pain symptoms and knee extension strength deficits in patellar tendinopathy, the relevance of these neural circuits to one another has not yet been investigated. Accordingly, it is reasonable to suggest this represents a major literature gap. Moreover, there are other gaps limiting insight for neurophysiological mechanisms driving patellar tendinopathy pathology, foremost among these being the temporal nature of motor neurophysiological changes. de Oliveira Silva et al. (2017) found there was an association between greater symptom duration and lower spinal excitability in people with patellofemoral pain, demonstrating motor neurophysiological mechanisms might progress over time. Although, it is not yet clear if similar occurs in patellar tendinopathy.

By addressing these literature gaps, important insight can be gained for the neurophysiological mechanisms driving patellar tendinopathy pathology. In particular, this knowledge might prove valuable to inform the development of more targeted interventions in future. The primary objective of this study was to establish whether the efficacy of neural circuits responsible for endogenous pain modulation (inhibition or facilitation) are associated with the excitability of neural elements that contribute to motor output in individuals with patellar tendinopathy. A secondary objective was to determine whether these pain modulatory circuits are associated with maximal isometric knee extension force, and if motor neurophysiological outcomes shared an association with symptom duration.

## Materials and methods

### Study design

This study used a cross-sectional observational design, with participants undergoing endogenous pain modulation assessment at an initial testing session, followed by motor neurophysiological assessment at a subsequent session (within one week). A larger cohort underwent pain modulation assessment (Vallance et al. 2025a, b), while a smaller subset of these participants also completed motor neurophysiological testing (Vallance et al. 2024); here, we report findings for the smaller nested subset of participants for whom data from both assessments were available. Notably, when examining motor neurophysiological outcomes in our prior publication, we observed lower knee extension maximal voluntary isometric force (MVIF) compared to asymptomatic athletic controls (Vallance et al. 2024)–given the current study draws from that cohort, we proceed with the assumption participants included in this study demonstrate lower knee extension MVIF typical of individuals with patellar tendinopathy (Obst et al. 2024).

Our study was approved by the Monash University Human Ethics Committee (MUHEC project ID: 19892), and we collected written informed consent from all participants. We used both the Strengthening the Reporting of Observational Studies in Epidemiology recommendations for cross-sectional and case-control studies, and the International Scientific Tendinopathy Consensus (ICON) recommended standards, to guide reporting in this study (Rio et al. 2020).

### Settings

Participants attended two separate testing sessions, each for a duration of two hours. The first session took place at the Monash Exercise Neuroplasticity Unit research laboratory, located at Monash University Peninsula Campus, Victoria, Australia, or at a private physiotherapy clinic in Melbourne, Victoria, Australia, and focused on endogenous pain modulation assessment. The second session was conducted at the same research laboratory specified above and focused on motor neurophysiological assessment.

### Participants

Between June 2020 and January 2023, we recruited participants from Melbourne-based sub-elite and community-based basketball and volleyball organisations. Additionally, we utilised advertisements on social media, as well as within Monash University departments. We initially contacted individuals who expressed interest in participating in our study via a telephone call, during which screening questions were answered.

Individuals passing the initial telephone screen were subsequently invited to attend the initial assessment, where a physical examination was conducted by a trained physiotherapist (PV) to confirm their eligibility. Participants were included if they were a basketball or volleyball athlete aged 18 years or above who met specific criteria to confirm the clinical diagnosis of patellar tendinopathy. Specifically, this diagnosis was based on self-reported pain that was localised to the inferior pole of the patella, which could be confirmed in the physical examination via palpation of the corresponding site (Millar et al. 2021). Additionally, participants were required to report features consistent with patellar tendinopathy, including (i) a gradual onset of symptoms that had lasted three months or longer; (ii) aggravation of pain with activities considered to load the patellar tendon (e.g., running, jumping, and/or walking downstairs); and (iii) heightened intensity of pain following periods of inactivity, such as upon waking, or following prolonged periods of sitting.

Participants also performed a single leg decline squat (SLDS), and were required to report a pain rating of at least 2/10 on an 11-point numerical rating scale (NRS; with 0 = no pain, and 10 = the worst pain imaginable) at 90^o^ knee flexion (Malliaras et al. 2015). Additionally, we used ultrasound imaging (Mindray M7, Shenzhen, China) to confirm the clinical diagnosis of patellar tendinopathy via detection of signs considered typical of tendinopathic change. These included hypoechoic regions within the proximal patellar tendon, and tendon thickening–notably, we employed a conservative threshold of >4 mm anterior-posterior diameter (Breda et al. 2021). This imaging was performed by an experienced physiotherapist, who is trained in the use of this technology (PV).

We excluded individuals if they were experiencing any painful condition at the time of testing (besides patellar tendon pain), or if they had experienced any painful condition within the prior six months to assessment that had lasted longer than one week, or that had been severe enough to require consultation with a health care professional. We also excluded individuals if they had received an injection (e.g., corticosteroid, platelet rich plasma or any other pharmaceutical agents) to the patellar tendon region or its surrounds within the six months prior, if they had a history of severe headaches or migraines, or had been diagnosed with type II diabetes, neurological conditions or an inherited connective tissue disorder. Further, we excluded anyone with metal in the cranial region, while all participants were also required to complete and fulfill the requirements of the TMS safety questionnaire (Rossi et al. 2011).

### Procedure

**Fig. 1:**
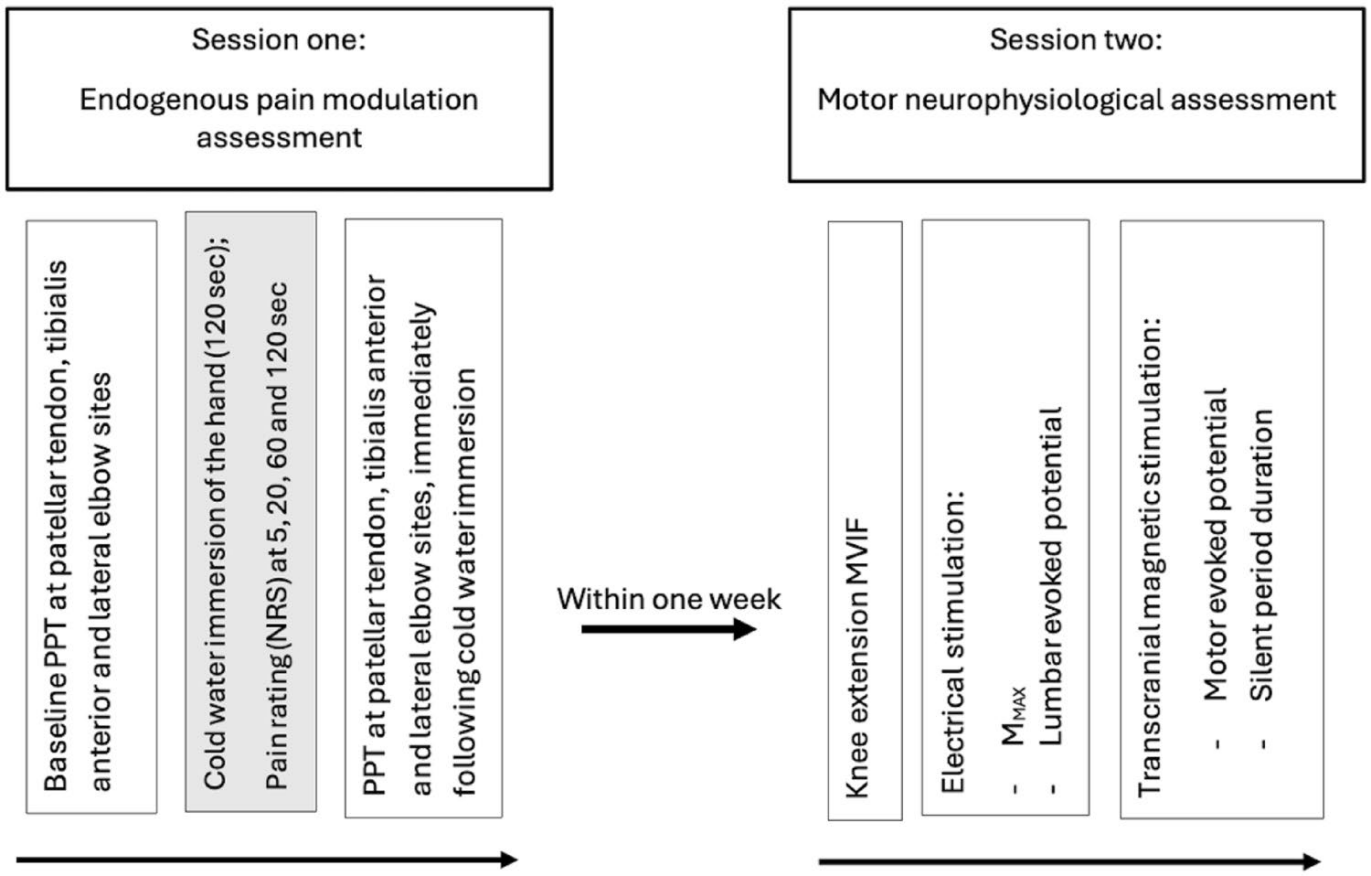
Participant flow through the first (endogenous pain modulation assessment) and second session (motor neurophysiological assessment). PPT pressure pain threshold, NRS numerical rating scale, MVIF maximum voluntary isometric force, M_MAX_ maximum compound action potential

At the initial testing session, we collected demographic and tendinopathy-specific details (e.g., duration of symptoms), and participants completed the VISA-P questionnaire (0–100, where 100 indicates no patellar tendon pain or functional impairments) (Visentini et al., 1998). Following this, we assessed the efficacy of neural circuits responsible for endogenous pain inhibition and facilitation, using an approach we have described in detail in a prior publication (Vallance et al. 2025a, b). A summary of endogenous pain modulation assessment procedure, and the motor neurophysiological assessment completed in the subsequent session, is provided in Fig. 1.

### Endogenous pain modulation assessment

We utilised a conditioned pain modulation protocol consistent with our prior investigation (Vallance et al. 2021), that conformed to the recommended approach (Lewis et al. 2012; Pud et al. 2009). We collected pressure pain thresholds (PPT; Newtons [N]) with a digital algometer (Force One FDIX, Wagner Instruments, Greenwich, USA) over the most painful site on the affected/most affected patellar tendon, the ipsilateral-side tibialis anterior muscle belly, and contralateral side lateral elbow epicondyle, before and immediately after the ipsilateral side hand (i.e., opposite to the elbow used for PPT assessment) was submerged in water cooled to induce pain. Sites were assessed in a computer-generated randomised order. We submerged participants hand in this water for a total duration of 120 s, up to the level of the wrist crease with fingers spread wide to avoid laminar warming (Tompra et al. 2016), with water temperature starting at 8 °C.

We aimed to evoke a minimum pain response of 3/10 NRS within 20 s of the hand being submerged; if this rating was not met, we planned to reduce water temperature by adding ice until the minimum pain target was achieved (at which point, water temperature was maintained). Notably, this rating was met in all participants without the need to add further ice. We asked participants to rate their pain at their hand on the NRS at five, 20, 60 and 120 s (Chimenti et al. 2020). Endogenous pain inhibition was quantified as percentage (%) change in PPT from before to immediately after painful cold-water immersion, and endogenous pain facilitation as the change in hand pain on the NRS while immersed in water, from the fifth to the 20th second (Chimenti et al. 2020). Pain inhibition was calculated at all three sites (Murphy et al. 2021). A number of steps were undertaken to enhance the consistency of endogenous pain measurement at either test settings (private physiotherapy clinic or research laboratory); namely, the same digital algometer and cold-water apparatus were used for all endogenous pain assessments, and this was carried out by the same trained physiotherapist (PV).

As the site utilised to apply the painful conditioning stimuli in our protocol was remote to the injured patellar tendon, our interpretation of endogenous pain modulation outcomes focused on the influence of supraspinal mechanisms on these responses. Specifically, this includes enhanced projections from supraspinal centres to facilitate or inhibit the transmission of nociceptive afferents at the spinal cord dorsal horn (Yarnitsky et al. 2014). This is as opposed to

segment specific effects, such as activity of nociceptors local to the injured patellar tendon that – if sensitised – could alter afferent input to influence endogenous pain response. Insight for the activity of such local neural elements would require a conditioning stimulus be applied to the affected area instead, although a limitation to that approach is that it would not be possible to delineate the influence of segment-specific from supraspinal mechanisms on the resulting endogenous pain response (Yarnitsky et al. 2014).

### Motor neurophysiological assessment

In the second testing session, we assessed participants knee extension MVIF, before measuring the excitability of neural elements that influence force production (i.e., motor neurophysiological outcomes). Our approach for this assessment has been described in detail in a prior publication (Vallance et al. 2024). Briefly, participants sat in our custom designed chair throughout this assessment, with their hips flexed to 90^o^ and their affected/most affected knee flexed to 60^o^. We attached the affected/most affected limb to an S-Type load cell (Sparkfun, Niwot, Colorado, USA) which had been calibrated to known values at the inferior tibia region – we attached the opposite end of the load cell to our chair, enabling knee extension force to be resisted isometrically.

Prior to electrode placement, we shaved the designated site to remove fine hair, applied an abrasive gel to remove dead skin cells, and sanitised the area using 70% isopropyl alcohol. We subsequently placed bipolar Ag–AgCl electrodes on the rectus femoris muscle to collect surface electromyography (sEMG), consistent with SENIAM guidelines (Hermens et al. 2000). We sampled and captured force signal (2048 Hz) using PowerLab 4/26, with a connected single-channel bridge amplifier (ADInstruments; Bella Vista, Australia) to allow force recordings to be synchronised with measures of neural element excitability. We amplified sEMG signal by the factor of 1000, and applied high- (13 Hz) and low-bandpass filtering (1000 Hz). Following collection, we analysed all signal recordings using PowerLab 4/26 software (ADInstruments, Bella Vista, Australia).

Following a familiarisation trial, participants performed two recorded knee extension maximal voluntary isometric force (MVIF) trials on their affected limb, interspersed with a rest period of at least 30 s. If maximal force achieved in these two trials differed by more than 5%, additional trial/s were performed until two trials were within this threshold (one of which being the highest force achieved). We considered the peak knee extension force achieved across all recorded trials to be the knee extension MVIF, and present this in its natural unit format (Kg/f) as well as standardised to body weight (Kg/Kg).

**Fig. 2:**
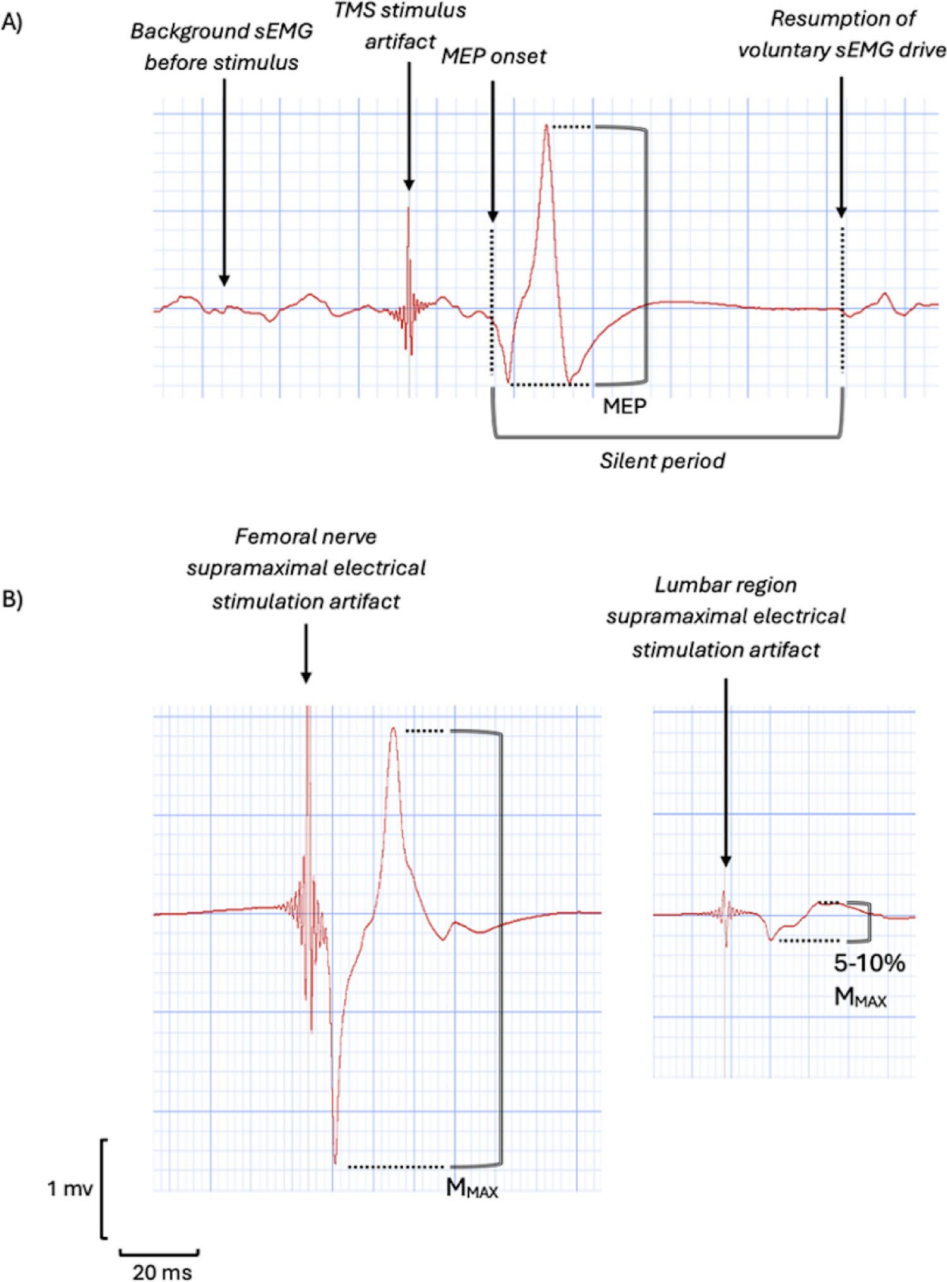
Visual representation of surface electromyographic (sEMG) data analysed, including **A** motor evoked potential (MEP; peak-to-peak sEMG following transcranial magnetic stimuli at 130% of active motor threshold, normalised to maximum compound action potential amplitude) and silent period duration (measured from onset of motor evoked potential to resumption of voluntary sEMG drive, and **B** maximum compound action potential (M_MAX_; peak-to-peak sEMG following electrical stimuli at a supramaximal intensity applied to the femoral nerve) and lumbar evoked potential (LEP; recorded as the electrical stimulator output required to elicit peak-to-peak sEMG equivalent to 5–10% of M_MAX_ sEMG, applied to the lumbar region). *mV* millivolts, *ms* milliseconds

We used a range of electrical stimulation and transcranial magnetic stimulation (TMS) techniques to assess the excitability of neural elements that contribute to knee extension motor output (Fig. 2). To apply electrical stimulation, we used a constant-current device (Digitimer DS7AH). Specifically, we assessed maximum compound action potential (M_MAX_) by delivering supramaximal electrical stimuli to the femoral nerve, and considered the highest peak-to-peak sEMG evoked with this approach to indicate the maximum muscle response (Rodriguez-Falces and Place 2018). We also used electrical stimulation to assess lumbar evoked potentials (LEPs), via application to the spinous processes of the first lumbar vertebra and eighth thoracic vertebra. We defined LEP as the electrical stimulation output (ESO; in mA) required to achieve sEMG amplitude within 5–10% of the M_MAX_ (Škarabot et al. 2019a, b). As LEP is considered to reflect the efficacy of efferent transmission between the corticospinal tract neurons and motoneurons (Škarabot et al. 2019a, b), a higher LEP ESO corresponds with lower spinal excitability (Vallance et al. 2024).

We used a Magstim 200^2^ device (Magstim Co., Ltd, Whitland, UK) to apply TMS, which was consistently administered over the M1 ‘hotspot‘ (Rothwell et al. 1999) while participants maintained 20% MVIC knee extension force. Notably, this hotpot was marked with a non-permanent marker to enable accurate application of stimuli. We delivered single-pulse TMS at 130% of active motor threshold to produce motor evoked potentials (MEP), which we calculated as the average peak-to-peak amplitude (mV) across 10 trials, expressed as a proportion of M_MAX_. Higher MEP amplitude is widely considered to indicate greater corticospinal excitability (Vallance et al. 2025a, b). We also calculated the period of sEMG inactivity following each single-pulse TMS-evoked MEP, which is recognised as the silent period. Specifically, we measured from the onset of the MEP to the reactivation of voluntary sEMG activity. The silent period is a marker of corticospinal inhibition, mediated by gamma-aminobutyric acid-B (GABA-B) receptor activity (Škarabot et al. 2019a, b).

### Statistical analysis

As this is a secondary analysis study, participants were enrolled to satisfy the requirements of our prior publication (Vallance et al. 2024). All analysis was completed using Statistical Package for the Social Sciences (SPSS; v.29; Chicago, USA). We inspected each outcome for normality using the Shapiro-Wilk test, and visual inspection of Q-Q plots. We present non-normally distributed data as median (interquartile range), and normally distributed data as mean (standard deviation). For correlational analysis, if one or both variables included were non-gaussian, we used Spearman’s Rho, while Pearson’s correlation coefficient was used if both variables being analysed were normally distributed. We investigated if endogenous pain facilitation or endogenous pain inhibition at the patellar tendon site shared association with motor neurophysiological outcomes (LEP ESO, MEP or silent period duration), or knee extension MVIF. Additionally, we investigated if symptom duration was association with any of these motor neurophysiological outcomes. To guide interpretation of correlations, 0.00–0.09 was considered the absence of a correlation, 0.10–0.39 a weak correlation, 0.40–0.69 moderate, 0.70–0.89 strong, and 0.90–1.00 a very strong correlation (Schober et al. 2018). Where any data were missing, cases were excluded from correlational analysis pairwise. To control for multiplicity, p values were adjusted using the Benjamini–Hochberg method as this approach is considered appropriate for exploratory analysis (Benjamini et al. 2001; Benjamini and Hochberg 1995).

To reduce the number of comparisons performed, we only included endogenous pain inhibition at the patellar tendon site in correlational analyses. To ensure this selection did not influence results, we performed sensitivity analysis to confirm the inclusion of pain inhibition response at the tibialis anterior or lateral elbow sites in correlations would not have produced different outcomes. Moreover, for identical reasoning, we only included knee extension MVIF in its raw unit in correlational analysis, so we performed sensitivity analysis to confirm inclusion of MVIF normalised to body weight (Kg/Kg) instead would not have influence results. Multiple comparisons were calculated for corresponding sensitivity analyses to enable fair comparison. We also tested the assumption that endogenous pain inhibition or facilitation response was not related to the intensity of hand pain experienced at five seconds immersion via correlational analysis, to ensure conclusions drawn for supraspinal influence on pain modulation (as opposed to peripheral input) were sound.

We used simple linear regression to examine the association between endogenous pain and motor neurophysiological outcomes, or knee extension MVIF, based on correlational analysis results (threshold for inclusion in models, *p* < 0.010). Where data were missing, we utilised group mean. Based on our modest sample and potential departures from normality, we incorporated bootstrapped regression coefficients and report bias-corrected and accelerated (BCa) confidence intervals (4000 resamples, with a fixed random seed for reproducibility; simple sampling method) (LaFlair et al. 2015). Holm-Bonferroni corrections were applied to p values associated with BCa intervals to control for multiple comparisons.

Residual plots were produced and visually inspected to check model assumptions were satisfied. Additionally, we computed diagnostics (Cook’s distance [D]) to assess for potential individual case leverage (indicated by Cook’s D > 1.0) and for sensitivity analysis – we re-ran each model after removing the observation with the highest Cook’s distance to determine potential influence of leverage; effect estimates and BCa intervals were compared to original models for stability. The significance for analysis associated with regression analysis was also set to *p* < 0.05.

## Results

We included nineteen basketball or volleyball athletes with patellar tendinopathy (mean age = 25.95 [SD 7.29] years, and BMI = 24.83 [SD 3.15] Kg/cm^2^) in this study. Almost all participants were male (18/19 [95%]), and experienced symptoms bilaterally (17/19 [89%]). Due to high individual stimulation threshold, TMS outcomes could not be collected in *n* = 4 participants (due to the capacity of the magnetic stimulator being exceeded), while LEP ESO could not be collected in *n* = 2 participants. A summary of tendinopathy specific, motor function, and neural element excitability outcomes is presented in Table 1.

**Table 1:** Summary of tendinopathy specific, endogenous pain modulation, motor function, and motor neurophysiological outcomes presented as mean (SD) unless otherwise specified

	Patellar tendinopathy (*n* = 19)
Pain and tendinopathy-specific outcomes	
Duration of symptoms, months^	36 (24, 60)
Pain with single leg decline squat, /10 NRS	5.4 (1.8)
Pain with hop, /10 NRS	2.5 (2.4)
Victorian Institute of Sport Assessment – Patellar, /100	59.6 (10.1)
Endogenous pain modulation response	
Endogenous pain facilitation, change in hand pain NRS (0 to 10) from five to 20 seconds hand immersion^	2.0 (1.5, 2.0)
Endogenous pain inhibition at the patellar tendon site, % PPT change from before to immediately after cold water immersion	11.8 (18.0)
Endogenous pain inhibition at the tibialis anterior site, % PPT change from before to immediately after cold water immersion	9.4 (18.5)
Endogenous pain inhibition at the lateral elbow epicondyle site, % PPT change from before to immediately after cold water immersion	13.0 (25.4)
Motor function	
Knee extension maximum voluntary isometric contraction, Kg	66.0 (16.3)
Normalised knee extension maximum voluntary isometric contraction, Kg / Kg	0.8 (0.2)
Motor neurophysiological outcomes	
Maximum compound action potential, amplitude (mV)	4.5 (1.2)
Lumbar evoked potential, electrical stimulator output *	301.4 (74.1)
Motor evoked potential, % maximum compound action potential ^#^	38.8 (16.5)
Silent period, duration (milliseconds) ^#^	86.4 (19.0)

^ indicates median (interquartile range); * indicates *n* = 17; ^#^ indicates *n* = 15

### Correlational analysis

*Relation between endogenous pain modulation response and motor neurophysiological outcomes* Supplementary appendix 1 summarises correlational analysis undertaken. We observed a moderate negative association between endogenous pain facilitation and knee extension MVIC (Kg/f) (rho = − 0.598, *p* = 0.007; *p*_adj_ = 0.042). A moderate positive association between endogenous pain facilitation and LEP ESO was also observed, although this did not meet the threshold for significance following adjustment for multiple comparisons (rho = 0.567, *p* = 0.013, *p*_adj_ = 0.052).

No associations were observed between endogenous pain inhibition and motor neurophysiological outcomes, or knee extension MVIF (*p* > 0.05).

**Fig. 3:**
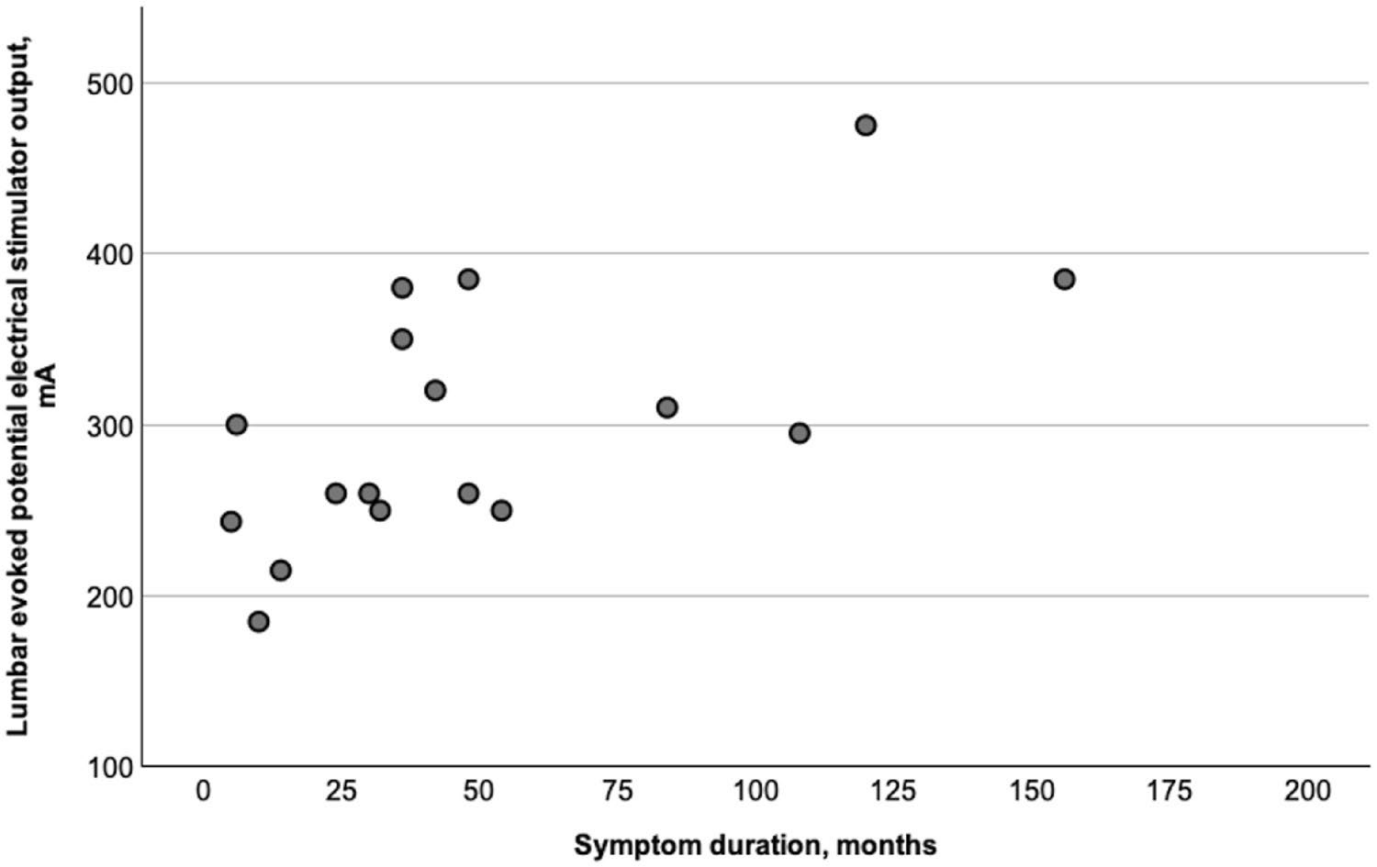
Relation between symptom duration in months and spinal excitability (lumbar evoked potential electrical stimulator output)

*Relation between motor neurophysiological outcomes and symptom duration *We observed a large positive association between symptom duration and LEP ESO (rho = 0.632, *p* = 0.006, *p*_adj_ = 0.042; Fig. 3). No other motor neurophysiological outcome was associated with symptom duration (*p* > 0.05).

### Simple linear regression

#### Model one

**Fig. 4:**
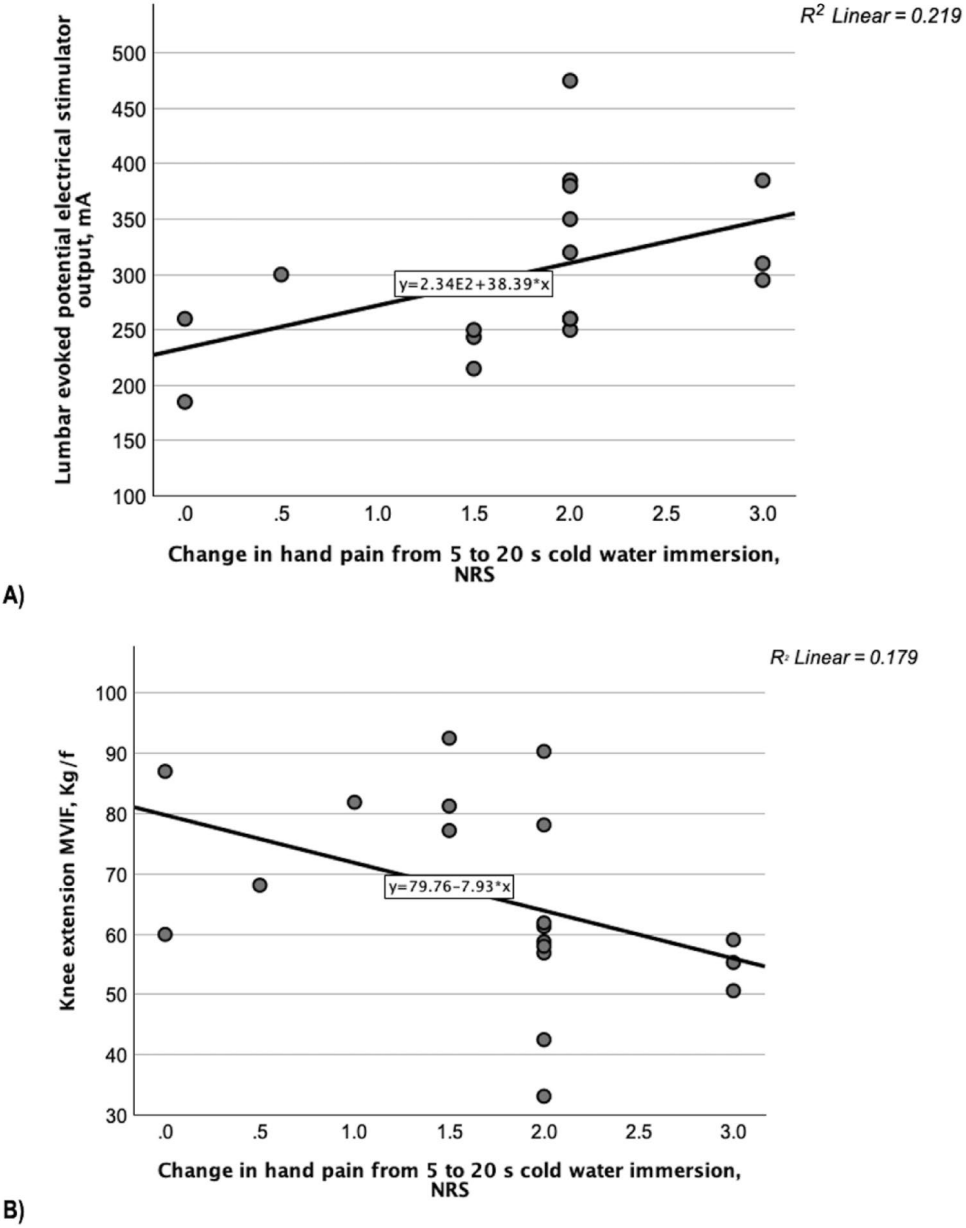
**A** Relation between endogenous pain facilitation (change in pain from 5 to 20 s hand immersion in cold water) and spinal excitability (lumbar evoked potential electrical stimulator output); **B** Relation between endogenous pain facilitation and maximal isometric knee extension strength (Kg/f)

A model that included endogenous pain facilitation as the predictor variable explained 22% of the variance in LEP (r^2^ = 21.9; *β* = 38.4 [BCa 95%CI 10.3 to 77.0], SEM = 14.8; *p* = 0.015, p_adj_ = 0.030; Fig. 4A).

#### Model two

A model that included endogenous pain facilitation as the predictor variable explained 18% of the variance in knee extension MVIF (r^2^ = 17.9; *β* = − 7.9 [BCa 95%CI − 15.1 to − 2.6], SEM = 3.7; p and *p*_adj_ = 0.031; Fig. 4B).

### Sensitivity analysis

#### Correlations

When normalised MVIF (Kg/Kg) was used instead of MVIF (Kg), the association with endogenous pain facilitation was no longer significant following adjustment for multiple comparisons (rho = − 0.489, *p* = 0.034, *p*_adj_ = 0.136). Inclusion of normalised MVIF did not alter the significance of other results, including non-significant correlations between normalised MVIF and endogenous pain inhibition (*r* = − 0.374, *p* = 0.114, *p*_adj_ = 0.228), and symptom duration (rho = − 0.413, *p* = 0.079, *p*_adj_ = 0.214). The inclusion of endogenous pain inhibition at the tibialis anterior, or lateral elbow epicondyle, in place of the patellar tendon site did not influence the significance of associations with motor neurophysiological outcomes (*p* > 0.05; supplementary appendix 2). The intensity of hand pain at five seconds cold water immersion was not associated with endogenous pain inhibition or facilitation (*p* > 0.05; supplementary appendix 3).

### Simple linear regression

#### Model one

Individual Cook’s D scores indicated model one estimates were not likely leveraged by individual cases (maximum Cook’s D = 0.214); the model remained stable when re-fitted without the participant with the highest Cook’s D score (r^2^ = 28.9; *β* = 35.2 [BCa 95%CI 11.3–66.2], SEM = 13.7; *p* and* p*_adj_ = 0.013).

#### Model two

Individual Cook’s D scores indicated model two estimates were not likely leveraged by individual cases (maximum Cook’s D = 0.158); the model remained stable when re-fitted without the participant with the highest Cook’s D score (r^2^ = 24.1; *β* = − 11.4 [BCa 95%CI − 19.5 to − 6.4], SEM = 3.5; *p* = 0.002, *p*_adj_ = 0.004).

## Discussion

In athletes with patellar tendinopathy, we found that greater endogenous pain facilitation predicted both reduced spinal excitability and lower knee extension MVIF. These results support the theory we recently proposed, whereby the function of neural circuits underlying endogenous pain facilitation might influence spinal excitability and, as a consequence, impair knee extension force output (Vallance 2025). Specifically, our results indicate an interaction could be present between enhanced transmission of nociceptive afferents at the spinal cord dorsal horn and increased excitability of inhibitory spinal interneurons. However, it must be stressed that causality cannot be inferred based on these results; rather, they might be best viewed as preliminary evidence for an association between these factors.

In our sensitivity analysis, the previously significant association between knee extension MVIF and endogenous pain facilitation was no longer present when absolute MVIF value was normalised to body weight. One potential explanation for this discrepancy is that absolute MVIF values might better reflect functional capacity in a ‘real-world’ setting, where factors beyond body weight – such as the excitability of neural elements that transmit signal to produce force - are more important to force production (Kasović et al. 2021; Vallance et al. 2025a, b). Moreover, normalising MVIF to body weight could obscure findings in an athletic cohort like ours. This is due to variable manner in which muscle and adipose tissue can comprise body weight, with the sum result retaining unclear relevance to force production capacity.

We did not detect a relationship between endogenous pain outcomes and the excitability of corticospinal neural elements involved in motor output. This is noteworthy, as previous research has established links between the presence of nociceptive afferents and alterations to the function of such motor circuits. For example, in individuals with knee osteoarthritis, reduced endogenous pain inhibition shared a relationship with decreased corticospinal inhibition (Tarrago et al. 2016). Our findings appear more consistent with those observed in individuals with patellofemoral pain. Specifically, de Oliveira Silva et al. (2017) found that higher self-reported pain over the preceding month was associated with lower Hoffman reflex (H-reflex) amplitude. The H-reflex reflects the excitability of alpha motoneurons, under conditions in which presynaptic inhibition and intrinsic motoneuron excitability are constant (Palmieri et al. 2004). Therefore, similar to LEPs, H-reflex amplitude would be impacted by spinal interneurons mediated inhibition, with lower amplitude corresponding with increased inhibition. Taken together, while drawing cautious parallels with patellofemoral pain, our findings raise the possibility interplay between pain and motor neurophysiological mechanisms not unlike quadriceps AMI documented elsewhere - rather than changes to corticospinal or M1 motor network excitability - may be a key contributor to knee extension strength deficits in athletes with patellar tendinopathy.

It is important to consider that there are notable differences between these conditions, e.g., in patellar tendinopathy, the source of nociceptive afferents is the patellar tendon itself (Millar et al. 2021), while in patellofemoral pain these are likely generated by knee joint-tissue (e.g., the synovial membrane) (Rice and McNair 2010). Despite these discrepancies, it is still reasonable to suggest that the term quadriceps AMI (or a term of similar understanding) might be suitable to describe this association between pain and motor neurophysiological mechanisms in athletes with patellar tendinopathy, given key characteristics of this phenomenon are satisfied. Specifically, there is upregulated/altered generation of nociceptive and non-nociceptive afferents (irrespective of the specific structure generating these) that is an apparent catalyst for reduced excitability of neural elements involved in motor function (Norte et al. 2024).

As was the case for the larger cohort from which the current studies participants were drawn (Vallance et al. 2025a, b), the endogenous pain facilitation response in our participants was highly variable. If a ‘typical’ or unaffected individuals pain facilitation response with our protocol is an increase of 1.8/10 NRS (Vallance et al. 2025a, b) - while also considering measurement accuracy (standard error of the mean = 0.4/10 NRS [calculated using hand-pain data collected at five seconds immersion in cold water]) - four of our participants (21%) experienced pain facilitation above this response, three (16%) below it, and 12 (63%) consistent with the typical response. Based on this variability, and the links established between endogenous pain facilitation and spinal excitability in our study, it could be suggested this interplay between neurophysiological mechanisms might not affect all individuals with patellar tendinopathy to the same extent. Rather, this neural mechanism might be most impactful on strength in those with heightened endogenous pain facilitation.

In this investigation, we also identified an association between longer symptom duration and lower spinal excitability. This is an intriguing result, particularly when considered alongside the typically long-persistence of patellar tendinopathy pathology (Visnes et al. 2024), and established relations between longer symptom duration and greater endogenous pain facilitation in this condition (Vallance et al. 2025a, b). The current study result is consistent with the findings of de Oliveira Silva et al. (2017) in patellofemoral pain. While limited for insight by a lack of prospective data (as is the case for our own study), de Oliveira Silva et al. (2017) promoted two potential hypotheses that could explain this relationship - lower spinal excitability might be the result of prolonged exposure to nociceptive afferent transmission at the spinal cord dorsal horn. Alternatively, they suggested lower spinal excitability might pre-exist the onset of pathology. We would suggest that if the second hypothesis were true, this would indicate the neurophysiological mechanisms underlying pain and motor function are independent of each other. Based on the associations detected between pain outcomes and spinal excitability in both investigations, that hypothesis appears less likely. Instead, it is more likely that people with either condition who suffer persistent pain symptoms develop lower spinal excitability across time, via the sensitising effect of ongoing afferent input on interneurons at the spinal dorsal horn (e.g., polysynaptic wide dynamic range neurons) (Rice and McNair 2010). However, such theory should be interpreted with caution given alternate hypotheses could explain this association. For example, it is also possible reducing spinal excitability could influence endogenous pain facilitation across time.

There has been an increased focus on identifying factors important to symptom severity, or treatment outcomes, in other persistent musculoskeletal conditions. For example, Hodges et al. (2025) describes ‘predictive biomarkers’, which are factors (or, a cluster of factors) that can be used to discriminate between people with low back pain who are expected to experience favourable or unfavourable outcomes with certain treatment approaches. Such an approach could prove valuable in patellar tendinopathy, given the suboptimal response of some individuals with current recommended rehabilitation (Breda et al. 2021). It is worth considering if endogenous pain facilitation could represent one such important biomarker in people with this condition. Although, it is important to acknowledge that while our findings indicate endogenous pain facilitation is at least relevant to knee extension strength deficits, longitudinal data are required to confirm any causation. Accordingly, future research could prioritise prospective investigations to establish if endogenous pain facilitation does in fact influence rehabilitation outcomes in people with patellar tendinopathy.

### Limitations

Participants included in this study consisted exclusively of athletes with patellar tendinopathy, who were predominately male, and this could have influenced our results. Sex differences have been identified for endogenous pain modulation response (Fillingim et al. 2009; Murphy et al. 2024; Popescu et al. 2010; van Wijk and Veldhuijzen 2010), as have differences between individuals who are active and sedentary (Mani et al. 2019; Naugle et al. 2017). Patellar tendinopathy is highly prevalent in sporting populations (in particular, basketball and volleyball sports) (Lian et al. 2005), while this condition is much more prevalent in males (Lian et al. 2005; Visnes and Bahr 2013; Zwerver et al. 2011). Therefore, our cohort with patellar tendinopathy should be considered reasonably representative of a typical population with this condition. We excluded participants with painful conditions besides patellar tendinopathy (current, or within the past six months) to minimise the presence of potential confounders. Such conditions would have impacted confidence when drawing conclusions the function of endogenous pain circuits shaped by presence of patellar tendinopathy pathology. However, it is expected that a proportion of individuals experiencing patellar tendinopathy might also suffer concurrent painful conditions. Consequently, generalisability might be limited to a subset of these athletes who are not also experiencing any additional painful conditions.

Our protocol applied a painful conditioning stimulus at a site remote to the injury region to enable clearer insight to be gained for the influence of supraspinal mechanisms on endogenous pain response. However, it is evident there was considerable variability for peripheral input between individuals (hand pain following 5 s immersion in cold water ranged between 1- and 6.5/10 NRS). It is possible differences in this peripheral input could have influenced endogenous pain response as well – this appears unlikely, given sensitivity analysis indicated pain intensity at five seconds immersion was not related to either pain facilitation or pain inhibition outcomes.

We measured sEMG activity over the rectus femoris muscle, which might have influenced results. It is possible motor neurophysiological outcomes might have differed had they been measured over alternate knee extensor muscles (e.g., vastus lateralis). There are several other factors that could have influenced results which were not accounted for in our analysis. For example, physiological factors have been demonstrated to influence pain neurophysiological outcomes in comparable musculoskeletal conditions (Othman et al. 2021). Additionally, physical activity levels within or outside of the athletes chosen sport, or any training load fluctuations, could have influenced this. However, their association with pain modulation outcomes are questionable (Lee et al. 2024).

Finally, we did not perform an a-priori power estimation. As a consequence, confidence in conclusions drawn from our results should be tempered, due to risk of type II error. This is particularly true for analyses performed using neural element excitability outcomes that were measured with TMS, given we could not collect these in a small number of participants (4/19, 21%). In our primary analysis, we utilised BCa bootstrapping to estimate intervals in regression models. This is an appropriate step to minimise sensitivity of leverage that could otherwise elevate risk of type 1 error, given the small sample. Moreover, this approach provides coverage for non-normality (LaFlair et al. 2015).

### Perspective

This study is the first to investigate the relation between neurophysiological mechanisms underlying pain symptoms and knee extension strength deficits in athletes with patellar tendinopathy. We found an association between higher endogenous pain facilitation and lower spinal excitability, as well as lower knee extension MVIF, indicating potential interplay between the underlying neurophysiological mechanisms. One plausible explanation for these results is that an interaction could be present between enhanced transmission of nociceptive afferents at the spinal cord dorsal horn and increased excitability of inhibitory spinal interneurons, although our findings should be considered preliminary and warrant further investigation.

## Supplementary Information

Below is the link to the electronic supplementary material.

Supplementary Material 1

Supplementary Material 2

## Abbreviations

AMI: Arthrogenic muscle inhibition.
ESO: Electrical stimulator output
GABA-B: Gamma-aminobutyric acid B
ICON: International consensus
LEP: Lumbar evoked potential
MEP: Motor evoked potential
M_MAX_: Maximum compound action potential
MVIF: Maximum voluntary isometric force
mV: Millivolts
NRS: Numerical rating scale
sEMG: Surface electromyography
SENIAM: Surface EMG for a non-invasive assessment of muscles
SLDS: Single leg decline squat
PPT: Pressure pain threshold
TMS: Transcranial magnetic stimulation
VISA-P: Victorian institute of sport assessment-patellar

## References

[CR1] Bannister K, Hughes S (2023) One size does not fit all: towards optimising the therapeutic potential of endogenous pain modulatory systems. Pain 164(1):e5–e9 35594517 10.1097/j.pain.0000000000002697 PMC9756434

[CR2] Benjamini Y, Hochberg Y (1995) Controlling the false discovery rate: a practical and powerful approach to multiple testing. J Roy Stat Soc: Ser B (Methodol) 57(1):289–300

[CR3] Benjamini Y, Drai D, Elmer G, Kafkafi N, Golani I (2001) Controlling the false discovery rate in behavior genetics research. Behav Brain Res 125(1–2):279–284 11682119 10.1016/s0166-4328(01)00297-2

[CR4] Breda SJ, Oei EH, Zwerver J, Visser E, Waarsing E, Krestin GP, de Vos R-J (2021) Effectiveness of progressive tendon-loading exercise therapy in patients with patellar tendinopathy: a randomised clinical trial. Br J Sports Med 55(9):501–509 33219115 10.1136/bjsports-2020-103403 PMC8070614

[CR5] Chimenti RL, Hall MM, Dilger CP, Merriwether EN, Wilken JM, Sluka KA (2020) Local anesthetic injection resolves movement pain, motor dysfunction, and pain catastrophizing in individuals with chronic Achilles tendinopathy: a nonrandomized clinical trial. J Orthop Sports Phys Ther 50(6):334–343 32349638 10.2519/jospt.2020.9242 PMC10016231

[CR6] Davi SM, Lepley AS, Denegar CR, DiStefano LJ, Edgar CM, Lepley LK (2020) Quadriceps inhibition after naturally occurring patellar tendon damage and pain. J Athl Train 55(6):608–614 32348153 10.4085/1062-6050-27-19 PMC7319745

[CR7] de Oliveira Silva D, Magalhães FH, Faria NC, Ferrari D, Pazzinatto MF, Pappas E, de Azevedo FM (2017) Vastus medialis Hoffmann reflex excitability is associated with pain level, self-reported function, and chronicity in women with patellofemoral pain. Arch Phys Med Rehabil 98(1):114–119 27422350 10.1016/j.apmr.2016.06.011

[CR8] Fillingim RB, King CD, Ribeiro-Dasilva MC, Rahim-Williams B, Riley JL III (2009) Sex, gender, and pain: a review of recent clinical and experimental findings. J Pain 10(5):447–485 19411059 10.1016/j.jpain.2008.12.001 PMC2677686

[CR9] Georgopoulos V, Akin-Akinyosoye K, Zhang W, McWilliams DF, Hendrick P, Walsh DA (2019) Quantitative sensory testing and predicting outcomes for musculoskeletal pain, disability, and negative affect: a systematic review and meta-analysis. Pain 160(9):1920–1932 31045746 10.1097/j.pain.0000000000001590 PMC6701980

[CR10] Hermens HJ, Freriks B, Disselhorst-Klug C, Rau G (2000) Development of recommendations for SEMG sensors and sensor placement procedures. J Electromyogr Kinesiol 10(5):361–374 11018445 10.1016/s1050-6411(00)00027-4

[CR11] Hernandez-Sanchez S, Hidalgo MD, Gomez A (2014) Responsiveness of the VISA-P scale for patellar tendinopathy in athletes. Br J Sports Med 48(6):453–457 23012320 10.1136/bjsports-2012-091163

[CR12] Hodges PW, Sowa G, O’Neill C, Vo N, Foster N, Samartzis D, Lotz J (2025) Development and application of predictive clinical biomarkers for low back pain care: recommendations from the ISSLS phenotype/precision spine focus group. Eur Spine J 34(4):1–10 39964488 10.1007/s00586-025-08720-4

[CR13] Kasović M, Štefan L, Štefan A (2021) Normative data for gait speed and height norm speed in ≥ 60-year-old men and women. Clin Interv Aging. 10.2147/CIA.S290071 33568903 10.2147/CIA.S290071 PMC7869711

[CR14] LaFlair GT, Egbert J, Plonsky L (2015) A practical guide to bootstrapping descriptive statistics, correlations, t tests, and ANOVAs. *Advancing quantitative methods in second language research*, *46*

[CR15] Lee S, Neogi T, McGinley B, Wang N, Law LF, Torabian KA, Aoyagi K, Stefanik JJ, Carlesso LC, Hausdorff JM (2024) Associations of pain sensitivity and conditioned pain modulation with physical activity: findings from the multicenter osteoarthritis study (MOST). Osteoarthr Cartil 32(8):982–989 10.1016/j.joca.2024.04.020 PMC11254545 38763431

[CR16] Lewis G, Rice D, McNair P (2012) Conditioned pain modulation in populations with chronic pain: a systematic review and meta-analysis. J Pain 13(10):936–944. 10.1016/j.jpain.2012.07.005 22981090 10.1016/j.jpain.2012.07.005

[CR17] Lian ØB, Engebretsen L, Bahr R (2005) Prevalence of jumper’s knee among elite athletes from different sports: a cross-sectional study. Am J Sports Med 33(4):561–567 15722279 10.1177/0363546504270454

[CR18] Malliaras P, Cook J, Purdam C, Rio E (2015) Patellar tendinopathy: clinical diagnosis, load management, and advice for challenging case presentations. J Orthop Sports Phys Ther 45(11):887–898 26390269 10.2519/jospt.2015.5987

[CR19] Mani R, Adhia DB, Leong SL, Vanneste S, De Ridder D (2019) Sedentary behaviour facilitates conditioned pain modulation in middle-aged and older adults with persistent musculoskeletal pain: a cross-sectional investigation. Pain Rep 4(5):e773 31875181 10.1097/PR9.0000000000000773 PMC6882573

[CR20] Millar NL, Silbernagel KG, Thorborg K, Kirwan PD, Galatz LM, Abrams GD, Murrell GA, McInnes IB, Rodeo SA (2021) Tendinopathy. Nat Rev Dis Primers 7(1):1–21 33414454 10.1038/s41572-020-00234-1

[CR21] Murphy MC, Rio EK, Chivers P, Debenham J, Docking SI, Travers M, Gibson W (2021) Do people with unilateral mid-portion Achilles tendinopathy who participate in running-related physical activity exhibit a meaningful conditioned pain modulation (CPM) effect: a pilot study. J Sci Med Sport 24(5):441–447 33187880 10.1016/j.jsams.2020.10.015

[CR22] Murphy MC, Mkumbuzi N, Keightley J, Gibson W, Vallance P, Riel H, Plinsinga M, Rio EK (2024) Conditioned pain modulation does not differ between people with lower-limb tendinopathy and nontendinopathy controls: a systematic review with individual participant data meta-analysis. J Orthop Sports Phys Ther 54(1):50–59 37854011 10.2519/jospt.2023.11940

[CR23] Naugle KM, Ohlman T, Naugle KE, Riley ZA, Keith NR (2017) Physical activity behavior predicts endogenous pain modulation in older adults. Pain 158(3):383–390 28187102 10.1097/j.pain.0000000000000769

[CR24] Norte GE, Sherman DA, Rush JL, Ingersoll CD, Bodkin SG, Snyder-Mackler L, Grindstaff TL, Burland JP, Hopkins JT, Blackburn T (2024) Advancing clinical evaluation and treatment of arthrogenic muscle inhibition: a need for validation and innovation. Am J Sports Med 52(12):NP34–NP36 39432392 10.1177/03635465241272410

[CR25] Nutarelli S, Lodi C, Cook JL, Deabate L, Filardo G (2023) Epidemiology of patellar tendinopathy in athletes and the general population: a systematic review and meta-analysis. Orthop J Sports Med 11(6):23259671231173659 37347023 10.1177/23259671231173659 PMC10280536

[CR26] Obst SJ, Peterson B, Heales LJ (2024) Maximal lower limb strength in patellar tendinopathy: a systematic review with meta-analysis. J Athl Train 59(2):159–172 37071550 10.4085/1062-6050-0662.22 PMC10895401

[CR27] Othman R, Jayakaran P, Swain N, Dassanayake S, Tumilty S, Mani R (2021) Relationships between psychological, sleep, and physical activity measures and somatosensory function in people with peripheral joint pain: a systematic review and meta-analysis. Pain Pract 21(2):226–261 32696604 10.1111/papr.12943

[CR28] Palmieri RM, Ingersoll CD, Hoffman MA (2004) The Hoffmann reflex: methodologic considerations and applications for use in sports medicine and athletic training research. J Athl Train 39(3):268 16558683 PMC522151

[CR29] Popescu A, LeResche L, Truelove EL, Drangsholt MT (2010) Gender differences in pain modulation by diffuse noxious inhibitory controls: a systematic review. Pain 150(2):309–318 20557999 10.1016/j.pain.2010.05.013

[CR30] Pud D, Granovsky Y, Yarnitsky D (2009) The methodology of experimentally induced diffuse noxious inhibitory control (DNIC)-like effect in humans. Pain 144(1):16–19 19359095 10.1016/j.pain.2009.02.015

[CR31] Rice DA, McNair PJ (2010) Quadriceps arthrogenic muscle inhibition: neural mechanisms and treatment perspectives. Semin Arthritis Rheum 40(3):250–266 19954822 10.1016/j.semarthrit.2009.10.001

[CR32] Rice DA, McNair PJ, Lewis GN, Dalbeth N (2014) Quadriceps arthrogenic muscle inhibition: the effects of experimental knee joint effusion on motor cortex excitability. Arthritis Res Ther 16(6):502 25497133 10.1186/s13075-014-0502-4 PMC4271337

[CR33] Rio EK, Mc Auliffe S, Kuipers I, Girdwood M, Alfredson H, Bahr R, Cook JL, Coombes B, Fu SN, Grimaldi A (2020) ICON PART-T 2019–International Scientific Tendinopathy Symposium Consensus: recommended standards for reporting participant characteristics in tendinopathy research (PART-T). Br J Sports Med 54(11):627–630 31519545 10.1136/bjsports-2019-100957

[CR34] Rodriguez-Falces J, Place N (2018) Determinants, analysis and interpretation of the muscle compound action potential (M wave) in humans: implications for the study of muscle fatigue. Eur J Appl Physiol 118:501–521 29282530 10.1007/s00421-017-3788-5

[CR35] Rossi S, Hallett M, Rossini PM, Pascual-Leone A (2011) Screening questionnaire before TMS: an update. Clin Neurophysiol 122(8):1686 21227747 10.1016/j.clinph.2010.12.037

[CR36] Rothwell JC, Hallett M, Berardelli A, Eisen A, Rossini P, Paulus W (1999) Magnetic stimulation: motor evoked potentials. The international federation of clinical neurophysiology. Electroencephalogr Clin Neurophysiol Suppl 52:97–103 10590980

[CR37] Schober P, Boer C, Schwarte LA (2018) Correlation coefficients: appropriate use and interpretation. Anesth Analg 126(5):1763–1768 29481436 10.1213/ANE.0000000000002864

[CR38] Škarabot J, Ansdell P, Brownstein CG, Thomas K, Howatson G, Goodall S, Durbaba R (2019a) Electrical stimulation of human corticospinal axons at the level of the lumbar spinal segments. Eur J Neurosci 49(10):1254–1267 30589956 10.1111/ejn.14321

[CR39] Škarabot J, Mesquita RN, Brownstein CG, Ansdell P (2019b) Myths and methodologies: how loud is the story told by the transcranial magnetic stimulation-evoked silent period? Exp Physiol 104(5):635–642 30830992 10.1113/EP087557

[CR40] Tarrago M, Deitos A, Brietzke AP, Vercelino R, Torres ILS, Fregni F, Caumo W (2016) Descending control of nociceptive processing in knee osteoarthritis is associated with intracortical disinhibition: an exploratory study [Observational Study]. Medicine 95(17):e3353. 10.1097/MD.0000000000003353 27124022 10.1097/MD.0000000000003353 PMC4998685

[CR41] Tompra N, van Dieën JH, Coppieters MW (2016) Central pain processing is altered in people with Achilles tendinopathy. Br J Sports Med 50(16):1004–1007. 10.1136/bjsports-2015-095476 26701922 10.1136/bjsports-2015-095476

[CR42] Vallance P, Crowley L, Vicenzino B, Malliaras P (2021) Contralateral mechanical hyperalgesia and altered pain modulation in men who have unilateral insertional Achilles tendinopathy: a cross-sectional study. Musculoskelet Sci Pract 52:102353 33636582 10.1016/j.msksp.2021.102353

[CR43] Vallance P, Malliaras P, Vicenzino B, Kidgell DJ (2024) Determining intracortical, corticospinal and alpha motoneurone excitability in athletes with patellar tendinopathy compared to asymptomatic controls. Scand J Med Sci Sports 34(2):1–11. 10.1111/sms.14579 10.1111/sms.14579 38332685

[CR44] Vallance P, Kidgell DJ, Vicenzino B, Malliaras P (2025a) Endogenous pain modulation is not different in basketball or volleyball athletes with patellar tendinopathy compared to asymptomatic athletic controls. Musculoskelet Sci Pract. 10.1016/j.msksp.2025.103280 39929089 10.1016/j.msksp.2025.103280

[CR45] Vallance P, Siddique U, Frazer A, Malliaras P, Vicenzino B, Kidgell DJ (2025b) Transcranial magnetic stimulation and electrical stimulation techniques used to measure the excitability of distinct neuronal populations that influence motor output in people with persistent musculoskeletal conditions: a scoping review and narrative synthesis of evidence. J Electromyogr Kinesiol. 10.1016/j.jelekin.2025.103011 40286533 10.1016/j.jelekin.2025.103011

[CR46] Vallance PM (2025) Neural deficits relevant to pain symptoms and functional impairments in individuals with Achilles or patellar tendinopathy. Monash University, Melbourne 10.1136/bjsports-2025-110865 41344866

[CR47] van Wijk G, Veldhuijzen DS (2010) Perspective on diffuse noxious inhibitory controls as a model of endogenous pain modulation in clinical pain syndromes. J Pain 11(5):408–419 20075013 10.1016/j.jpain.2009.10.009

[CR48] Visentini PJ, Khan KM, Cook JL, Kiss ZS, Harcourt PR, Wark JD, Group V (1998) The VISA score: an index of severity of symptoms in patients with jumper’s knee (patellar tendinosis). J Sci Med Sport 1(1):22–28 9732118 10.1016/s1440-2440(98)80005-4

[CR49] Visnes H, Bahr R (2013) Training volume and body composition as risk factors for developing jumper’s knee among young elite volleyball players. Scand J Med Sci Sports 23(5):607–613 22260424 10.1111/j.1600-0838.2011.01430.x

[CR50] Visnes H, Bache-Mathiesen LK, Yamaguchi T, Gilhuus HP, Algaard KRH, Hisdal E, Bahr R (2024) Long-term prognosis of patellar tendinopathy (Jumper’s Knee) in young, elite volleyball players: tendon changes 11 years after baseline. Am J Sports Med 52(13):3314–3323 39439271 10.1177/03635465241284648 PMC11542324

[CR51] Yarnitsky D, Granot M, Granovsky Y (2014) Pain modulation profile and pain therapy: between pro-and antinociception. Pain 155(4):663–665 24269491 10.1016/j.pain.2013.11.005

[CR52] Zwerver J, Bredeweg SW, Van Den Akker-Scheek I (2011) Prevalence of Jumper’s knee among nonelite athletes from different sports: a cross-sectional survey. Am J Sports Med 39(9):1984–1988 21737835 10.1177/0363546511413370

