# Supplementary material for: Greater endogenous pain facilitation is associated with lower spinal excitability and maximal knee extension strength deficits in athletes with patellar tendinopathy
Source: Eur J Appl Physiol. 2026 Feb 25;126(6):3437–50. doi: 10.1007/s00421-026-06166-0 (PMC13287251; doi:10.1007/s00421-026-06166-0)
Supplement: Supplementary file 1 — Supplementary Material 1 [file 421_2026_6166_MOESM1_ESM.docx]

**Supplementary appendix 1.** Summary of associations between endogenous pain modulation response and symptom duration, and motor function and motor neurophysiological outcomes.

| Motor function / motor neurophysiological outcome | Endogenous pain modulation response | | Symptom duration |
| --- | --- | --- | --- |
|  | **Endogenous pain inhibition** | **Endogenous pain facilitation** |  |
| Maximum voluntary isometric force, Kg  *n = 19* | r = -0.377,  p = 0.111  p_adj_ = 0.222 | **rho = -0.598,**  **p = 0.007**  **p_adj_ = 0.042 *** | rho = -0.386,  p = 0.102  p_adj_ = 0.222 |
| Lumbar evoked potential, electrical stimulator output  *n = 17* | r = -0.064,  p = 0.807  p_adj_ = 0.854 | rho = 0.589,  p = 0.013  p_adj_ = 0.052 | **rho = 0.632,**  **p = 0.006**  **p_adj_ = 0.042 *** |
| Motor evoked potential  *n = 15* | r = -0.093,  p = 0.742  p_adj_ = 0.854 | rho = 0.193,  p = 0.490  p_adj_ = 0.653 | rho = 0.052,  p = 0.854  p_adj_ = 0.854 |
| Silent period  *n = 15* | r = 0.347  p = 0.205  p_adj_ = 0.351 | rho = 0.210  p = 0.453  p_adj_ = 0.653 | rho = 0.454,  p = 0.089  p_adj_ = 0.222 |

**Supplementary appendix 2.** Summary of sensitivity analyses; associations between motor function and motor neurophysiological outcomes and endogenous pain inhibition, using tibialis anterior or lateral elbow epicondyle site response in place of the patellar tendon site, in the patellar tendinopathy group.

| Motor function / motor neurophysiological outcome | Endogenous pain inhibition | |
| --- | --- | --- |
|  | Tibialis anterior site | Lateral elbow site |
| Maximum voluntary isometric force, Kg  *n = 19* | r = 0.234,  p = 0.334  p_adj_ = 0.535 | r = 0.286,  p = 0.235  p_adj_ = 0.410 |
| Lumbar evoked potential, electrical stimulator output  *n = 17* | r = 0.449,  p = 0.070  p_adj_ = 0.204 | r = -0.198,  p = 0.447  p_adj_ = 0.588 |
| Motor evoked potential  *n = 15* | r = 0.224,  p = 0.423  p_adj_ = 0.535 | r = -0.324,  p = 0.239  p_adj_ = 0.410 |
| Silent period  *n = 15* | r = 0.252  p = 0.365  p_adj_ = 0.535 | r = -0.034  p = 0.906  p_adj_ = 0.906 |

**Supplementary appendix 3.** Summary of sensitivity analyses; associations between the intensity of hand pain on a numerical rating scale following five seconds immersion in cold water, and endogenous pain inhibition at the patellar tendon site and endogenous pain facilitation.

|  | Endogenous pain modulation response | |
| --- | --- | --- |
|  | **Endogenous pain inhibition** | **Endogenous pain facilitation** |
| Hand pain following five seconds immersion in cold water, numerical rating scale | r = 0.334,  p = 0.162 | rho = -0.246,  p = 0.310 |
